# Supplementary material for: Comparison of Methods for Incorporating Related Data When Developing Clinical Prediction Models: A Simulation Study
Source: Biom J. 2026 Aug 27;68(5):e70166. doi: 10.1002/bimj.70166 (PMC13520826; doi:10.1002/bimj.70166)
Supplement: Supplementary file 2 [file BIMJ-68-e70166-s001.zip › third submission code review - R2/reproducibility_supplement/results/case_study/FIGURE8(a)-Bootstrap Apparent Metrics.pdf]

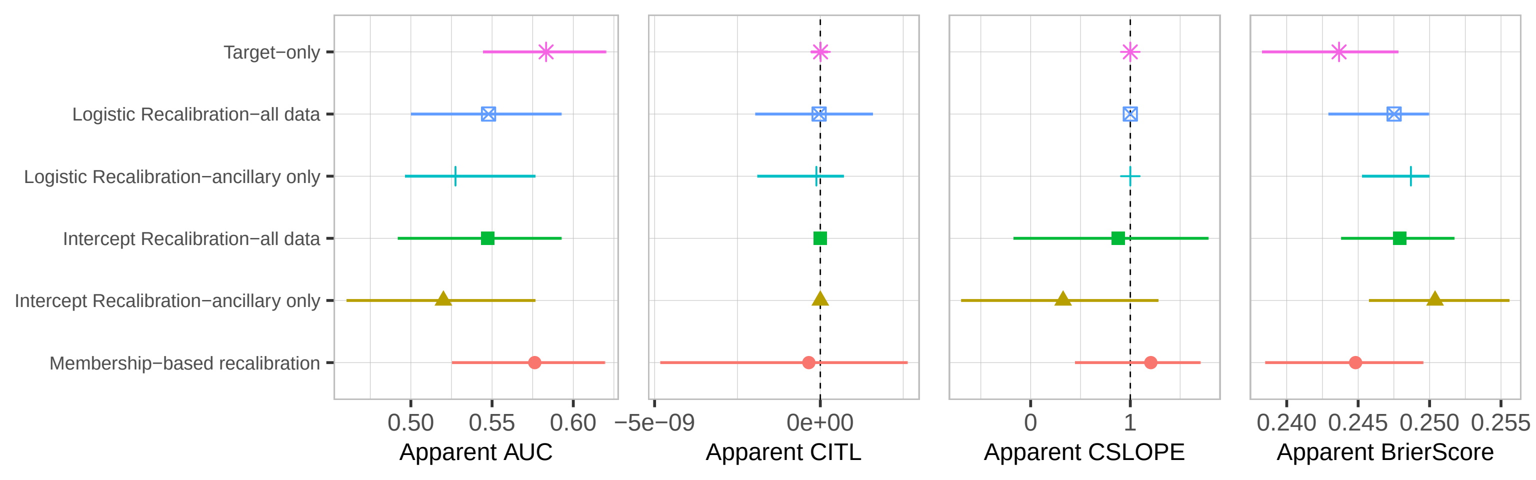

Target−only
Logistic Recalibration−all data
Logistic Recalibration−ancillary only
Intercept Recalibration−all data
Intercept Recalibration−ancillary only
Membership−based recalibration
0.50
0.55
0.60
Apparent AUC
−5e−09
0e+00
Apparent CITL
0
1
Apparent CSLOPE
0.240
0.245
0.250
0.255
Apparent BrierScore
